# Rupture of the Perineum

**Published:** 1902-09-06

**Authors:** 


### RUPTURE OF THE PERINEUM.

In a lecture delivered at the London Polytechnic, Dr. Gow[1] made some remarks on rupture of the perineum which may convey some comfort to those who have had the misfortune to preside over the occurrence of this by no means unfrequent accident. There is a common idea, that the occurrence of rupture of the perineum is a slur on the skill of the practitioner, and the indirect result of that belief is that in many cases where the perineum is torn it is not sewn up again, so as to avoid exposing the supposed fault. But I think, he says, there has not yet been invented any device which will prevent the perineum rupturing in many cases. "You will meet with people who will tell you—and I have met with many—that in the cases they attend the perineum never ruptures. That on the face of it is really absurd." The perineum ruptures in a certain proportion of cases of primiparæ. That it does not often rupture in multiparæ is due to the fact that the majority of these people do not possess a perineum, and therefore rupture does not occur for the best of reasons. When people say they do this or that, and the consequence is that their patients do not have ruptured perineums, it seems to imply that if there is a rupture there must be some fault. As a matter of fact, whether the perineum ruptures or not depends infinitely more on the perineum than on the medical attendant. "Bearing in mind, then, that rupture of the perineum is frequently inevitable in normal delivery, there can be nothing to be ashamed of from the fact that it occurs. And, if one does not boast of one's ability to prevent it, there is no reason why one should not sew it up when it does occur." That is the great point.

[1] The Clin. Jour., Aug. 27.

